# Comment on ; Post-infectious glomerulonephritis presenting as acute renal failure in a patient with Lyme disease

**DOI:** 10.12861/jrip.2014.11

**Published:** 2013-11-14

**Authors:** Hamid Nasri

**Affiliations:** ^1^Department of Nephrology, Division of Nephropathology, Isfahan University of Medical Sciences, Isfahan, Iran

**Keywords:** Glomerulonephritis, IgA nephropathy, Infectious diseases

**Implication for health policy/practice/research/medical education:**

Most cases of IgA nephropathy (IgAN) are idiopathic, however, various diseases are associated with IgAN. Recent findings have suggested that IgAN may be a consequence of infections, possibly due to an abnormal IgA-mediated immune response against microbial antigens with formation of nephrotoxic immune complexes. It is possible that removal of the causative organism from circulation by appropriate treatment leads to improvements in kidney function through increased clearance of immune-complexes, thus leading to the resolution of IgA nephropathy.

The interesting article, published by Rolla et al, had some points needs more description. They reported a post-infectious glomerulonephritis secondary to Borrelia burgdorferi infection in a 61-year old man (1). The diagnosis of glomerulonephritis was IgA nephropathy, through immunofluorescence study by significant IgA deposits in the mesangial area and negative C1q deposits. They concluded that, Borrelia burgdorferi is a possible cause of post-infectious glomerulonephritis in humans as it is in dogs. While most cases of IgA nephropathy (IgAN) are idiopathic, various diseases are associated with IgAN (1). Between July 2000 and February 2009, 21 cases of post infectious glomerulonephritis were evaluated by Wen et al. in Taiwan. They found, glomerular IgA dominant or co-dominant deposition was more frequently seen in atypical pattern (2). Various findings have suggested that IgAN may be a consequence of infections, possibly due to an abnormal IgA-mediated immune response against microbial antigens with formation of nephrotoxic immune complexes (3). It is possible that removal of the causative organism from circulation by appropriate treatment leads to improvements in kidney function through increased clearance of immune-complexes, thus leading to the resolution of IgA nephropathy (4-7). Recently we had reported a 28-year-old man was admitted because of fever and abdominal pain. Primary evaluations showed right kidney pyonenphrosis. In spite of placing a nephrostomy tube, fever continued. Repeated CT revealed focal pyelonephritis. Additionally, peripheral blood smear suggested malaria. Anti-malarial agents were initiated and right nephrectomy was conducted. One year after recovery from malaria, a persistent rise in serum creatinine was detected (creatinine before disease was 0.8 mg/dl which reached to 1.6 mg/dl). A left renal biopsy showed mild mesangial proliferation with dominant IgA deposits (>2+) in immunofluorescence study while C1q was not deposited (8). The impression was immunoglobulin A nephropathy with M_1_E_0_S_0_T_0_ (Oxford classification). The patient was prescribed a combination of low dose prednisolone and angiotensin converting enzyme inhibitor. Six months after treatment serum creatinine decreased from 1.6 mg/dl to 1.3 mg/dl and urine sediment became normal. Our findings suggest that malaria infection might be associated with IgA nephropathy. IgAN is the most common primary glomerulonephritis through the world. It is defined by mesangial cell proliferation, expansion of the extracellular matrix, and predominant IgA deposition in the mesangium region (8).

In summary, the clinical pattern and the final outcome of involved patients may vary depending on the severity of renal lesions detected at kidney biopsy. Indeed, one of the potent point of this case, was classification of IgAN by Oxford system, which best shows the severity of six morphologic lesions of this classification and will give an exact interpretation for treatmental medalities (4-7).

## Author’s Contribution

HN is the single author of the manuscript.

## Conflict of interests

None to declare.

## Ethical considerations

Ethical issues (including plagiarism, data fabrication, and duplicate publication) have been completely observed by the authors.

## Funding/Support

No financial support by any institution.
